# Evaluation of an attention deficit hyperactivity disorder (ADHD) assessment & treatment service

**DOI:** 10.1192/bjo.2021.887

**Published:** 2021-06-18

**Authors:** Chan Nyein, David Oyewole

**Affiliations:** 1 Northwick Park Mental Health Unit; 2 Northwick Park Mental Health Unit, CNWL Adult ADHD Clinic

## Abstract

**Aims:**

The Central and North West London NHS Foundation Trust ADHD clinic offers diagnosis and medication stabilisation for adults with ADHD, in preparation for discharge back to GP for continued prescribing and monitoring. Referral waiting time is shortened by efficiently managing the service and soon transfer of care to GP whilst referrals have been increasingly accepted years on years. A snap shot service evaluation was made to understand characteristics of service exploring its strength and areas to improve.

**Method:**

All 115 patients offered in March and April 2019 for an ADHD specialist assessment were sampled from the new electronic patient record SystmOne in use since 1st March 2019.

Data were collected for

Male & Female ratio

Age range distribution

Clinical Commissioning Group referral source

Clinic attendance characteristics

ADHD diagnosis, sub-types and psychiatric comorbidity

ADHD Medication prescribed

FP10 Prescription duration by prescribers

Patient data were anonymously encoded into Microsoft Excel Sheet for sorting, counting, summating and illustrating into tables and pie charts.

**Result:**

The male & female ratio of the sample was 6:5 and nearly half were in age range 20-29 years. Majority were referred from Westminster and West London Clinical Commissioning Groups.

107 patients completed the assessment, of which 106 were diagnosed as having an adult ADHD.

22% of follow-up clinics were cancelled or not attended (DNA) by patients. The majority of the patients (62%) required 1-2 follow-ups before transfer to GP, whilst 8% did not require or want follow-ups either already being on ADHD medication, not wanting medication or having lost to reviews. Only 3% require six or more follow-ups.

Majority were reviewed after two- to five-week prescription, the peak being four-weekly.

91% of completion to GP were discharged on ADHD medication, majority being singly on Elvanse (48%) and Concerta XL (25%). Discharge without ADHD medication was due to concerns for its addiction, preference on non-medication treatment, intolerance of medication adverse effect or mental health priority treatment.

**Conclusion:**

Collaboration with GPs for their pre-treatment physical health screening facilitated prompt prescribing initiation on assessment with most discharges taken place after 1-2 follow-ups, enabling service turn-over with short waiting time (6-9 months in 2018/2019). Service expansion for increasing referral uptake is probably feasible from this baseline by appointing additional sessional clinicians and further efficiency management on clinic scheduling & DNA with a target majority likely requiring 1-2 follow-ups with average four-weekly prescribing.

